# Association between parafunctional behaviors, clinical diagnoses, psychosocial factors and pain widespreadness in Finnish TMD pain patients in tertiary care

**DOI:** 10.22514/jofph.2025.074

**Published:** 2025-12-12

**Authors:** Arvid Iljin, Ilona Assila, Ritva Näpänkangas, Tuija Teerijoki-Oksa, Mimmi Tolvanen, Kirsi Sipilä

**Affiliations:** ^1^Research Unit of Population Health, University of Oulu, 90570 Oulu, Finland; ^2^Medical Research Center Oulu, Oulu University Hospital and University of Oulu, 90220 Oulu, Finland; ^3^Department of Oral and Maxillofacial Diseases, Turku University Hospital, 20520 Turku, Finland; ^4^University of Oulu, 90570 Oulu, Finland

**Keywords:** Biopsychosocial, DC/TMD, Oral parafunctions, Pain drawing, Temporomandibular disorders, Widespread pain

## Abstract

**Background**: To evaluate the association of oral parafunctions with clinical DC/TMD (Diagnostic Criteria for Temporomandibular Disorders) Axis I diagnoses, Axis II biopsychosocial assessment and pain widespreadness in TMD pain patients in tertiary care. **Methods**: 197 TMD pain patients were clinically examined and responded to DC/TMD OBC (Oral Behaviour Checklist) and Axis II comprehensive instruments. Patients were divided into Pain Drawing (PD) profile subgroups: PD-1 = head/face pain; PD-2 = head and neck/shoulder regional pain; PD-3 = widespread pain. Using the Graded Chronic Pain Scale 2.0 assessing pain-related intensity/interference, the patients were classified into TMD subtypes 1–3. Associations of frequent sleep bruxism (4–7 times per week) and daytime clenching (most/all of the time) with explanatory variables were evaluated with Independent Samples Kruskal-Wallis and chi-square tests and pairwise comparisons were made with Mann-Whitney U-test with Bonferroni correction. **Results**: Frequent sleep bruxism was reported by 46.2% and daytime clenching by 67.5% of the participants. Sleep bruxism and daytime clenching associated significantly with muscle-related TMD diagnoses. Sleep bruxism and daytime clenching were significantly associated with anxiety (GAD-7, General Anxiety Disorder-7) subgroups, the highest prevalence being in the most severe subgroups. Frequent sleep bruxism was reported more by participants in TMD subtype 2 as well as those in PD-2 and PD-3 profile subgroups, with significant differences between PD-1 *vs.* PD-2 and between PD-1 *vs.* PD-3. **Conclusions**: Oral parafunctions are associated with muscle-related TMD diagnoses, anxiety symptoms and wider body pain, which should be considered in the assessment, treatment planning and personalized care of TMD pain patients.

## 1. Introduction

Temporomandibular disorders (TMD) refer to pain and/or dysfunction of the temporomandibular joints (TMJs), masticatory muscles, and associated structures. The most common symptoms of TMD are pain in the jaw and masticatory muscles, limited jaw movements, and TMJ sounds [1]. In addition, patients with TMD commonly report headache, facial and ear pain, and comorbid pain symptoms, including neck, shoulder and/or back pain, or fibromyalgia [2].

Parafunctional behaviors include sleep bruxism, defined as a masticatory muscle activity during sleep (characterized as rhythmic or tonic activity), and awake bruxism, which is masticatory muscle activity during wakefulness and characterized by repetitive or sustained tooth contact and/or by bracing or thrusting of the mandible [3]. Singing, talking for a long time (in customer service or teaching, for example) and chewing gum are also parafunctional activities of the masticatory system, among others [4]. Oral parafunctions have shown to relate with the incidence of TMD [5, 6]. Further, both sleep and awake bruxism have been shown to be associated with pain-related diagnoses of TMD [7, 8, 9, 10]. Bruxism can also be linked to other pains in the body, widespread pain, and headache [11, 12, 13]. On the contrary, studies based on objective measurements (*i.e.*, electromyography, polysomnography) have not found any association or even a negative relationship between sleep bruxism and TMD [14].

The current criteria for TMD, DC/TMD (Diagnostic Criteria for Temporomandibular Disorders), are applicable for both research and clinical use [15]. The DC/TMD criteria consist of Axis I and Axis II, of which Axis I elucidates patient’s somatic diagnoses using DC/TMD Symptom Questionnaire and clinical examination protocol, while Axis II comprises biopsychosocial instruments for assessment of pain-related, jaw function and psychological and psychosocial factors [15]. The DC/TMD Axis II instruments include Graded Chronic Pain Scale (GCPS version 2.0), Patient Health Questionnaires (PHQ-4, PHQ-9 and PHQ-15), Generalized Anxiety Disorders (GAD-7), and Jaw Function Limitation Scale (JFLS-8 and JFLS-20). The Oral Behavior Checklist (OBC) inquires parafunctions of the masticatory system assessing, including sleep and awake bruxism, and other parafunctional behaviors. Pain Drawing (PD) is an instrument for visualizing and assessing pain locations, distribution, and widespreadness of TMD pain and comorbid pain problems [15]. A Finnish translation of the DC/TMD criteria, DC/TMD-FIN (Finnish translation), has been developed [16], and its applicability and validity have recently been evaluated in a multicenter study in Finland [17].

Among TMD pain patients, different biopsychosocial profiles have been shown based on pain-related intensity/interference and pain widespreadness [17]. Using the GCPS 2.0, a study on Finnish tertiary care referral TMD pain patients showed that those in the most severely compromised TMD pain patients (TMD subtype 3) experienced biopsychosocial symptoms and comorbid pains [17], as well as more widespread pain marked in DC/TMD Pain Drawings significantly more often than patients with mild and moderately compromised burden related to TMD (TMD subtypes 1 and 2) [18].

Parafunctional behaviors have been shown to relate to psychological distress [12]. Further, physical and depression symptoms have shown to be associated with self-reported waking-state oral parafunctional behaviors, assessed by using the OBC [10, 19]. However, the relationship between oral parafunctions and psychosocial factors as well as psychosocial profiling has been studied only little with valid DC/TMD instruments among tertiary care TMD pain patients. Also, studies with the sample size matching this study are scarce. Further studies are needed to provide information on the association between psychological factors and bruxism, in order to plan individualized treatment plan for TMD pain patients.

The aim of this study was to evaluate the association of oral parafunctions with clinical DC/TMD Axis I diagnosis and Axis II biopsychosocial assessment, *i.e.*, pain-related intensity/interference, depression and physical symptoms, anxiety and pain widespreadness in TMD pain patients in tertiary care, based on a multi-center study. It can be hypothesized that oral parafunctions are associated with muscle-related TMD diagnoses and with psychosocial burden as well as with widespread pain.

## 2. Material and methods

The study sample consisted of 197 patients with temporomandibular disorder (TMD) pain, including 158 females and 39 males, with a mean age of 43.3 years (Standard deviation (SD) = 16.7, range 17–83 years). These patients were referred for treatment at tertiary care facial pain clinics located in Helsinki University Hospital, Kuopio University Hospital, Oulu University Hospital, and Turku University Hospital in Finland, between July 2015 and March 2019. All patients aged 17 years or older and having a clinical diagnosis of TMD were included in the study. Those not fulfilling these inclusion criteria were excluded. Participation in the study was voluntary, and all subjects provided written informed consent. The study was approved by the Ethics Committee of the Hospital District of Southwest Finland (approval number: 74/1082/2015).

Prior to the implementation of the study, the Finnish versions of the DC/TMD Symptom Questionnaire, Axis I protocol, and all Axis II instruments underwent a comprehensive translation and cultural adaptation process. This process was conducted by the International Research Diagnostic Criteria for Temporomandibular Disorders (RDC/TMD) Consortium in accordance with the Guidelines for Establishing Cultural Equivalency of Instruments [20].

### 2.1 Axis I clinical diagnostics

The clinical DC/TMD Axis I diagnostics was based on the DC/TMD Symptom Questionnaire, the DC/TMD standardized clinical examination protocol and Axis I decision trees, and the diagnostic criteria table [16]. The diagnoses included myalgia, myofascial pain, myofascial pain with referral, arthralgia, headache attributed to TMD, disc dislocations with/without reduction, degenerative joint disease, and TMJ luxation. Multiple diagnoses were allowed.

The clinical TMD examinations were performed according to the DC/TMD-FIN [16]. The clinical examiners had been calibrated against reference standard examiner [17].

### 2.2 Axis II instruments

The patients received the questionnaires to be filled in at home, and these were assessed for accuracy with the treating clinicians at the first assessment visit. The DC/TMD-FIN Axis II questionnaires included assessment of TMD pain-related intensity/interference (GCPS 2.0), symptoms of depression (PHQ-9), anxiety (GAD-7), physical symptoms (PHQ-15), head and body pain localization (Pain Drawing, PD) and parafunctional activities (OBC) (DC/TMD-FIN, https://inform-iadr.com/wp-content/uploads/2024/03/DC-TMD-Finnish-Assessment-Instruments_revised-2021_06_23.pdf) [16].

According to the GCPS 2.0, the GCPS grades I–IV were determined as follows according to Orhbach and Knibbe [21, 22].

Furthermore, GCPS grade II was subdivided into GCPS II-Low and GCPS II-High. Based on GCPS grades the TMD subtypes 1–3 were formed [17].

Depression symptoms were assessed using the PHQ-9-FIN instrument, which includes 9 questions. Each item is rated on a scale from 0 to 3, where 0 indicates “not at all” and 3 indicates “nearly every day”. Based on the total score, depression severity was classified as follows: a score of ≥5 indicates mild depression, ≥10 moderate depression, ≥15 moderately severe depression, and ≥20 severe depression.

Anxiety symptoms were measured using the GAD-7-FIN instrument, which consists of 7 questions. Each item is rated on a scale from 0 to 3, where 0 means “not at all” and 3 means “nearly every day”. Based on the total score, anxiety severity was classified as follows: a score of ≥5 indicates mild anxiety, ≥10 moderate anxiety, and ≥15 severe anxiety.

Physical symptoms were assessed using the PHQ-15-FIN instrument, which includes 15 questions. Each item is rated on a scale from 0 to 2, where 0 means “not bothered” and 2 means “bothered a lot during the past four weeks”. Based on the total score, symptom severity was classified as follows: a score of ≥5 indicates low symptom severity, ≥10 medium symptom severity, and ≥15 high symptom severity.

In PDs, patients were asked to indicate all different pain sites and locations in the whole body and facial/head areas by shading each pain site [16]. The evaluation of PDs has been presented in detail in Iljin *et al*. [18]. The patients were classified into three pain drawing profile subgroups [23] PD-1 (1) = pain located only in facial and head area; PD-2 (2) = regional pain in the trigeminal-cervical region (including head and neck/shoulder regions); PD-3 (3) = widespread pain (including local/regional and multiple bodily pain sites outside the areas of PD-1 and 2). Each patient was only classified into one PD profile subgroup. In total, 196 TMD pain patients answered and/or indicated their pain sites and locations on DC/TMD Axis II Pain Drawings (PD). One patient did not have a PD included in the DC/TMD-FIN assessments.

The OBC includes 21 questions to assess different types and the frequency of parafunctional behaviors. The first two questions relate to activities during sleep (sleep bruxism); “How often do you do each of the following activities, based on the last month?”.

(1) Clench or grind teeth when asleep, based on any information you may have.

(2) Grind teeth together during waking hours.

(3) Clench teeth together during waking hours.

The response options for Q1 were “none of the time”, “<1 night/month”, “1–3 nights/month”, “1–3 nights/week” and “4–7/nights/week”, and for Q2 and Q3, “none of the time”, “a little of the time”, “some of the time”, “most of the time” and “all of the time”. The OBC sum score was calculated as the sum of score of each question (from 0, “none of the time” to 4, “4–7-nights/week” or “all of the time”) [23].

In PHQ-9, PHQ-15 and GAD-7 sum scores, missing values were replaced with the mean value of other items. If there were missing values for more than the following limits, the response was considered missing for the instrument: GCPS 2.0; Characteristic Pain Intensity (CPI) 0 items (n = 6), pain-related activity interference: 1 item (n = 8), PHQ-9: 3 items (n = 5), PHQ-15: 5 items (n = 4), GAD-7: 2 items (n = 6). The number of missing values for OBC items were 8 for Q1, 5 for Q2, and 3 for Q3 and Q4.

The questions of the OBC that were left blank were interpreted as point 0 (none of the time). If more than two of the 21 questions of the OBC were missing, no sum score for the patient was calculated (n = 16). Pain drawing was interpreted as missing data in case of its absence (n = 1).

### 2.3 Sociodemographic data

The DC/TMD-FIN Demographics included sociodemographic background data [16]. For the analysis, marital status was dichotomized as married/cohabiting *vs.* single (divorced, separated, widowed, or never married). Level of education was dichotomized as lower (basic education/high school/vocational school) *vs.* higher (university of applied sciences/university/Master of Arts). Working status was dichotomized as employed (working outside home/at home) *vs.* unemployed (unemployed/student/retired/on disability/retired due to sickness/sick leave/in rehabilitation).

### 2.4 Statistical analyses

Responses to the OBC questions 1 (sleep bruxism) and 2 (jaw pressure during sleep) were dichotomized as follows: “non-frequent sleep bruxism” (none of the time/<1 night per month/1–3 nights per month/1–3 nights per week) *vs.* “frequent” sleep bruxism (4–7 nights per week). Responses to OBC 4 (daytime clenching) were dichotomized as “non-frequent” none/a little/some of the time *vs.* “frequent” (most/all of the time).

The associations of sleep bruxism and daytime clenching (as dichotomized) with sociodemographic data, DC/TMD Axis I diagnoses, TMD subtypes, and PHQ-9, PHQ-15, GAD-7 and PD profile subgroups were evaluated using crosstabulations and Chi-square tests, and the effect sizes were calculated using Cramer’s V tests.

The OBC questions 1 and 4 were also used continuous variables. The differences in mean scores of questions 1 and 4 and total sum score of OBC between all different subgroups were evaluated using Kruskal-Wallis test. In pairwise comparisons of sleep bruxism, daytime clenching and OBC sum scores between PD profile subgroups and TMD subtypes Mann-Whitney U-test was used. In pairwise comparisons after Bonferroni correction, the statistical significance was set at *p * < 0.017.

## 3. Results

Based on the OBC, 46.2% of the participants reported frequent sleep bruxism, and 67.5% reported frequent pressure on the jaw during sleep (Table 1). Frequent daytime grinding was reported by 5.1% and frequent daytime clenching by 19.8% of the participants. There was a statistically significant gender difference in daytime clenching (*p* = 0.032), female patients reporting more daytime clenching than males (81% *vs.* 67%).

**Table 1.** Frequencies (percentages) of self-reported parafunctions, based on Oral Behavior Checklist (OBC) questions 1–4 by gender in the study sample of 197 TMD pain patients.

		n (%)	n_female_ (%)	n_male_ (%)	p*
Sleep bruxism		197 (100.0)	158 (100.0)	39 (100.0)	
Q1: Clenching and grinding when asleep		189 (95.6)	150 (94.9)	39 (100.0)	
	None of the time	46 (23.4)	30 (19.0)	16 (41.0)	0.103
	<1 night/mon	9 (4.6)	7 (4.4)	2 (5.1)	
	1–3 nights/mon	11 (5.6)	9 (5.7)	2 (5.1)	
	1–3 nights/wk	32 (16.2)	27 (17.1)	5 (12.8)	
	4–7 nights/wk	91 (46.2)	77 (48.7)	14 (35.9)	
Q2: Pressure on the jaw during sleep		192 (97.5)	153 (96.8)	39 (100.0)	
	None of the time	15 (7.6)	11 (7.0)	4 (10.3)	0.932
	<1 night/mon	8 (4.1)	7 (4.4)	1 (2.6)	
	1–3 nights/mon	10 (5.1)	8 (5.1)	2 (5.1)	
	1–3 nights/wk	26 (13.2)	20 (12.7)	6 (15.4)	
	4–7 nights/wk	133 (67.5)	107 (67.7)	26 (66.7)	
	Daytime clenching	197 (100.0)	158 (100.0)	39 (100.0)	
Q3: Grinding teeth together during waking hours		194 (98.5)	155 (98.1)	39 (100.0)	
	None of the time	109 (55.3)	92 (58.2)	17 (43.6)	0.132
	A little of the time	43 (21.8)	31 (19.6)	12 (30.8)	
	Some of the time	32 (16.2)	25 (15.8)	7 (17.9)	
	Most of the time	9 (4.6)	7 (4.4)	2 (5.1)	
	All of the time	1 (0.5)	0 (0.0)	1 (2.6)	
Q4: Clenching teeth together during waking hours		194 (98.5)	155 (98.1)	39 (100.0)	
	None of the time	40 (20.3)	27 (17.1)	13 (33.3)	0.032
	A little of the time	37 (18.8)	28 (17.7)	9 (23.1)	
	Some of the time	78 (39.6)	67 (42.4)	11 (28.2)	
	Most of the time	32 (16.2)	29 (18.4)	3 (7.7)	
	All of the time	7 (3.6)	4 (2.5)	3 (7.7)	

*Chi-square test.

Sociodemographic data by sleep bruxism/daytime clenching are presented in Table 2. There was no significant associations between sleep bruxism/daytime clenching and age or gender. Participants with single marital status reported significantly more sleep bruxism (*p* = 0.048) and daytime clenching (*p* = 0.034) than married/cohabiting participants. Furthermore, employed participants reported significantly more sleep bruxism than unemployed participants (56.4% *vs.* 40.7%, *p* = 0.032). Similarly, employed participants had significantly higher mean OBC score than unemployed participants (*p* = 0.012).

**Table 2.** Distributions (percentages) of sociodemographic variables by frequent sleep bruxism (Q1) and daytime clenching (Q4) in the study sample of 197 TMD pain patients based on Oral Behavior Checklist (OBC).

Characteristics		Sleep bruxism		Daytime clenching		OBC score	
		n (%)	p*	n (%)	p*	Mean (SD)	p**
Gender							
	Male	14 (35.9)	0.086	6 (15.4)	0.411	26.9 (10.0)	0.099
	Female	77 (51.3)		33 (21.3)		29.5 (8.6)	
Marital status							
	Married/cohabiting	50 (42.4)	0.048	18 (14.9)	0.034	27.9 (8.7)	0.141
	Single	38 (57.6)		19 (27.5)		30.5 (8.9)	
Education							
	Lower	50 (45.5)	0.292	22 (19.3)	0.801	28.9 (9.5)	0.810
	Higher	40 (53.3)		16 (20.8)		29.2 (8.1)	
Working status							
	Employed	53 (56.4)	0.032	20 (21.1)	0.690	30.7 (8.4)	0.012
	Unemployed	37 (40.7)		18 (18.8)		27.3 (9.3)	

*Chi-square test. **Kruskal-Wallis test. OBC: Oral Behaviour Checklist; SD: Standard deviation.

Sleep bruxism was significantly associated with muscle-related TMD (*p* = 0.029). Of the sub-diagnoses, myofascial pain with referral was significantly associated with daytime clenching as well (*p* = 0.009). Additionally, participants with muscle-related TMD had significantly higher mean OBC scores than those with no diagnosis (*p* = 0.010) (Table 3).

**Table 3.** Distributions (percentages) of DC/TMD Axis I diagnoses by sleep bruxism and daytime clenching in the study sample of 197 TMD pain patients.

Characteristics		Sleep bruxism (%)			Daytime clenching (%)			Mean OBC score (SD)		
		No (n = 98)	Yes (n = 91)	p*	No (n = 155)	Yes (n = 39)	p*	No dg	Dg	p**
Muscle-related TMD (n = 140)		67.3	81.3	0.029	73.5	79.5	0.445	26.0 (9.4)	29.9 (8.6)	0.010
	Myalgia (n = 136)	65.3	79.1	0.035	71.0	79.5	0.286	26.6 (9.3)	29.8 (8.7)	0.033
	Myofascial pain with referral (n = 89)	38.8	56.0	0.017	43.2	66.7	0.009	27.8 (8.9)	30.2 (8.8)	0.050
	Headache attributed to TMD (n = 75)	30.6	49.5	0.008	37.4	51.3	0.115	27.2 (9.3)	31.5 (7.8)	<0.001
Joint-related TMD (n = 175)		89.8	91.2	0.741	89.7	92.3	0.621	28.5 (8.4)	29.0 (9.0)	0.943
	Arthralgia (n = 136)	72.4	71.4	0.876	72.3	69.2	0.708	28.7 (9.0)	29.0 (9.0)	0.748
	Disc dislocations with reduction (n = 33)	19.4	15.4	0.469	17.4	17.9	0.938	28.9 (9.1)	29.1 (8.5)	0.777
	- With intermittent locking (n = 3)	1.0	2.2	0.518	1.9	0.0	0.381	28.9 (9.0)	29.3 (2.9)	0.991
	Disc dislocations without reduction with limited opening (n = 21)	9.2	13.2	0.382	12.9	7.7	0.368	28.7 (9.2)	30.4 (5.9)	0.448
	- Without limited opening (n = 54)	27.6	29.7	0.747	28.4	25.6	0.732	28.4 (9.1)	30.4 (8.4)	0.190
	Degenerative joint disease (n = 39)	24.5	16.5	0.174	21.3	15.4	0.411	29.5 (8.7)	26.9 (9.5)	0.103

*Chi-square test. **Kruskal-Wallis test. OBC: Oral Behaviour Checklist; TMD: Temporomandibular Disorders; SD: Standard deviation; Dg: Diagnosis.

Sleep bruxism (*p* = 0.016) and daytime clenching (*p* = 0.005) were significantly associated with anxiety (GAD-7) subgroups, the highest prevalence being in the most severe subgroups (Table 4). Furthermore, significantly higher mean OBC scores were found in participants with higher subgroups of GAD-7 (*p* = 0.008) and PHQ-15 (*p* = 0.034) than in those in the lower subgroups. When using the OBC Q1 (sleep bruxism) and Q4 (daytime clenching) as continuous variables, their associations with GAD-7 subgroups were significant (Table 5).

**Table 4.** Distributions (percentages) of frequent sleep bruxism and daytime clenching by biopsychosocial variables (Patient Health Questionnaire-9 and 15, and General Anxiety Disorder-7, GAD-7) and Pain Drawing (PD) subgroups in the study sample of 197 TMD pain patients based on Oral Behavior Checklist (OBC).

Characteristics			Sleep bruxism		Daytime clenching	
		Total n	n (%)	p* (effect size**)	n (%)	p* (effect size**)
TMD subtype						
	Subtype 1	84	35 (41.7)	0.034 (0.194)	14 (16.5)	0.406 (0.125)
	Subtype 2	22	16 (72.7)		5 (22.7)	
	Subtype 3	73	35 (47.9)		20 (26.3)	
PHQ-9 subgroup						
	No/mild	141	69 (46.3)	0.526 (0.083)	28 (18.3)	0.484 (0.087)
	Moderate	23	12 (52.2)		6 (26.1)	
	Moderate-severe	13	8 (61.5)		4 (28.6)	
PHQ-15 subgroup						
	No	24	11 (44.0)	0.492 (0.115)	5 (20.0)	0.265 (0.145)
	Low severity	71	34 (45.3)		14 (18.2)	
	Medium severity	47	22 (45.8)		7 (14.3)	
	High severity	36	22 (59.5)		12 (30.8)	
GAD-7 subgroup						
	No	104	44 (40.7)	0.016 (0.236)	13 (11.7)	0.005 (0.259)
	Mild	46	28 (58.3)		16 (32.7)	
	Moderate	15	8 (50.0)		4 (25.0)	
	Severe	12	10 (83.3)		5 (38.5)	
PD-subgroup						
	Subgroup 1	58	18 (30.5)	0.006 (0.235)	7 (11.7)	0.084 (0.160)
	Subgroup 2	65	39 (55.7)		15 (20.8)	
	Subgroup 3	57	33 (55.9)		17 (27.8)	

*Chi-square test. **Cramer’s V test. TMD: Temporomandibular Disorders; PHQ: Patient Health Questionnaires; GAD: General Anxiety Disorder; PD: Pain Drawing.

**Table 5.** Mean (SD, standard deviation) scores for sleep bruxism (Q1) and daytime clenching (Q4), and total OBC score, by biopsychosocial variables (Patient Health Questionnaire-9 and 15, and General Anxiety Disorder-7, GAD-7) and Pain Drawing (PD) subgroups in the study sample of 197 TMD pain patients based on Oral Behavior Checklist (OBC).

			Sleep bruxism			Daytime clenching			OBC score		
		n	Mean (SD)	Median (Q1–Q3)	p*	Mean (SD)	Median (Q1–Q3)	p*	Mean (SD)	Median (Q1–Q3)	p*
TMD subtype											
	1	80	2.4 (1.7)	3 (0–4)	0.103	1.6 (1.1)	2 (1–2)	0.297	28.0 (9.2)	29 (21–33)	0.263
	2	22	3.1 (1.6)	4 (2.5–4)		2.0 (0.8)	2 (1.75–2.25)		30.0 (6.2)	30.5 (25.75–33.25)	
	3	69	2.6 (1.6)	3 (1–4)		1.7 (1.2)	2 (1–3)		30.0 (9.6)	31 (22.5–36.5)	
PHQ-9											
	No/mild	141	2.5 (1.6)	3 (0–4)	0.390	1.6 (1.1)	2 (1–2)	0.187	28.3 (8.5)	29 (22–34.5)	0.217
	Moderate	23	2.6 (1.6)	4 (1–4)		2.8 (1.6)	2 (2–3)		31.7 (9.6)	31 (27–37)	
	Moderate-severe	13	3.2 (1.5)	4 (3–4)		3.2 (1.5)	2 (1–3)		30.1 (10.6)	32 (23–40)	
PHQ-15											
	No	24	2.1 (1.9)	3 (0–4)	0.172	1.3 (1.2)	1 (0–2)	0.256	25.8 (10.9)	25.5 (18–31.75)	0.034
	Low	71	2.5 (1.6)	3 (1–4)		1.5 (1.0)	2 (1–2)		28.0 (7.6)	29 (22–32)	
	Medium	47	2.5 (1.7)	3 (0–4)		1.8 (1.0)	2 (1–2)		30.0 (8.1)	31 (23–36)	
	High	36	3.2 (1.3)	4 (3–4)		1.9 (1.1)	2 (1–3)		31.6 (9.6)	32 (26.25–38.5)	
GAD-7											
	No	104	2.4 (1.7)	3 (0–4)	0.012	1.4 (1.0)	2 (1–2)	0.014	27.2 (8.0)	27.5 (21–32.75)	0.008
	Mild	46	3.0 (1.4)	2 (1–3)		1.9 (1.2)	4 (2.3–4)		30.0 (8.5)	31 (23.5–36)	
	Moderate	15	2.6 (1.7)	3.5 (0.5–4)		2.0 (1.0)	2 (2–2.8)		32.7 (10.5)	32 (29–40)	
	Severe	12	3.6 (1.2)	4 (4–4)		2.1 (1.1)	2 (1–3)		34.3 (10.6)	35.5 (29.5–41.5)	
PD-subgroup											
	1	58	2.0 (1.7)	2 (0–4)	0.002	1.4 (1.0)	2 (0–2)	0.104	26.0 (9.2)	25.5 (19–32.25)	0.005
	2	65	3.0 (1.4)	4 (2–4)		1.7 (1.1)	2 (1–2)		30.9 (8.3)	32 (25.5–36.5)	
	3	57	2.8 (1.7)	4 (1–4)		1.8 (1.1)	2 (1–3)		30.0 (8.8)	30 (24.5–35)	

*Kruskal-Wallis test. TMD: Temporomandibular Disorders; PHQ: Patient Health Questionnaires; GAD: General Anxiety Disorder; PD: Pain Drawing; SD: standard deviation.

TMD subtypes are associated significantly with sleep bruxism (*p* = 0.034). Participants in TMD subtype 2 reported significantly more frequent sleep bruxism than participants in TMD subtype 1 or 3 (Table 4). There were no significant associations of sleep bruxism or daytime clenching with pain intensity (CPI), disability days and disability score by pain interference. In pairwise comparisons, a statistically significant difference, after Bonferroni adjusted *p*-value being set at *p* = 0.017, was found between TMD subtype 1 and 2 patients (*p* = 0.010) in frequency of sleep bruxism. When using the OBC Q1 (sleep bruxism) and Q4 (daytime clenching) as continuous variables, their associations with TMD subtypes were not significant (Table 5).

PD-profile distribution associated significantly with sleep bruxism (*p* = 0.002). More than half of the TMD pain patients in PD-2 and PD-3 profiles reported sleep bruxism, whereas the proportion in PD-1 profile was lower (31%). OBC scores differed significantly between PD profile subgroups (*p* = 0.005), being highest among PD-2 and lowest among PD-1 TMD pain patients. In pairwise comparisons, statistically significant differences were found between PD-1 and PD-2 profile subgroup patients (*p* = 0.004) and between PD-1 and PD-3 profile subgroup patients (*p* = 0.006) in frequency of sleep bruxism. A significant difference was also found between PD-1 and PD-2 profile subgroup patients (*p* = 0.002) when comparing OBC sum scores. Mean score of OBC Q1 differed significantly between PD profile subgroups, being highest in PD-2. There were no significant differences in OBC Q4 between PD profile subgroups (Table 5).

The missing OBC sum score due to non-response was analyzed; there were no significant differences between non-respondents *vs.* respondents in TMD subtype, or PHQ-9, PHQ-15, GAD-7, or PD subgroups (Chi-square test).

## 4. Discussion

The present study evaluated the prevalence of oral parafunctions by means of the OBC questionnaire in DC/TMD criteria and their association with clinical Axis I diagnoses and biopsychosocial background factors such as pain-related interference, depression and somatization symptoms, anxiety, and other body pains and widespreadness of pain. In the OBC, sleep bruxism was evaluated through questions 1–2 and daytime clenching through questions 3–4. The prevalence of both frequent sleep bruxism (Q1, 46.2%) and daytime clenching (Q4, 19.8%) was high among TMD pain patients. The present study showed that sleep bruxism was associated with muscle-related TMD diagnoses, anxiety symptoms, and wider body pain distribution (*i.e.*, neck and shoulder region pain and widespread pain in multiple bodily pain sites).

In the present study especially sleep bruxism, but also daytime clenching, was associated with muscle-related TMD diagnoses. Sleep bruxism was significantly associated with TMD sub-diagnoses, *i.e.*, with myalgia, myofascial pain with referral and TMD-attributed headache, whereas daytime clenching was associated with myofascial pain with referral only. This is in accordance with a population-based study by Fernandes *et al*. [4], who concluded that parafunctions during both sleep and waking are associated with painful TMD. The present findings are also partly in line with the earlier studies on TMD patients, showing that sleep bruxism was associated with pain-related TMD [24, 25] and higher distress levels, GCPS and OBC [25]. Furthermore, it has been reported that the risk for TMD pain increases with the frequency of parafunctional activities [26]. The present study showed that both sleep and awake bruxism are associated with pain-related TMD diagnoses. Earlier studies have also noted that the risk for TMD pain did not differ in separate reports of sleep or awake bruxism [27, 28]. Additionally, the presence of both awake and sleep bruxism may interact additively with painful TMD [27]. In addition to using sleep and awake bruxism as classified, the present study used the OBC summary score, indicating the overall frequency of parafunctional activity. The OBC sum score showed parallel associations with most of the variables. The sum score has been used to evaluate the risk of TMD; a score from 25 to 62 indicated 17 times more often having a risk factor for TMD [29]. The mean OBC sum scores exceeded this limit in the present study. In clinical practice it is important to question the patients about both sleep and awake bruxism when TMD pain is present.

In this study, the presence of anxiety symptoms (based on GAD-7) was significantly associated with frequent sleep bruxism and daytime clenching; patients with higher subcategories of anxiety and physical symptoms had significantly higher mean OBC scores than participants with milder symptoms. These results are partly supported by a cross-sectional study by Xu *et al*. [30], where 537 TMD patients were assessed using similar DC/TMD instruments (OBC, GAD-7, JFSL and PHQ-9). In their study, the OBC scores were significantly associated with anxiety symptoms, jaw functional limitation and depression symptoms. Also, a study from 54 TMD patients and 46 controls showed that the presence of anxiety symptoms was associated with OBC total score and sleep-related oral behaviors, based on DC/TMD [19]. The results encourage adding clinical applications such as psychoeducation or cognitive-behavioral interventions in the treatment of TMD patients with anxiety symptoms.

The present study did not show any significant association of depression symptoms with sleep bruxism, daytime clenching or mean OBC score, although a tendency for higher frequency of sleep bruxism was noted in the more severe subgroups of depression symptoms. In the literature there are controversial findings concerning this association; some studies have found associations between oral behaviors and depression [19], whereas others have not found any significant association [31]. The present results may suggest that anxiety symptoms have a stronger role in self-reported bruxism than depression symptoms do. However, it should be noted that the present study had limitations due to the distribution of PHQ-9 subgroups. The majority of patients (n = 141) were classified in the PHQ-9 no/mild category, whereas the proportions in the more severe categories were smaller, which is why the statistical power was weak. Therefore, a larger sample would be needed to better evaluate this association. DC/TMD PHQ-9 measures only depressive mood at the time questionnaire is answered. In this study TMD pain patients’ exact medical background, that is, possible diagnosed depression and medication, is unknown and can affect the results.

The present study showed an association between sleep bruxism and TMD subtypes; participants with TMD subtype 2 with moderate pain intensity/interference reported significantly more sleep bruxism than participants with TMD subtype 1 or 3. In the literature, there exist only few studies investigating the association between parafunctions and TMD subtyping/GCPS grading. On the contrary to the present results, a study from 1220 TMD patients showed that perceived sleep bruxism was most prevalent in the groups in the most severe GCPS grades (III and IV, corresponding to TMD subtype 3) [32]. The results of the present study indicate that sleep bruxism may be an important background factor, especially in this patient group having moderate pain intensity/interference. An earlier study from the present sample showed that patients in TMD subtype 2 had an intermediate biopsychosocial burden compared to TMD subtypes 1 and 3 [17]. It is important to consider bruxism among other TMD-related factors to prevent the risk for chronic TMD in TMD subtype 2. In TMD subtype 3, the role of sleep bruxism might be less important than the psychosocial burden, as indicated by the presence of depression and anxiety symptoms as well as physical symptoms [17]. In clinical practice it is important to clarify TMD pain patients’ pain state by using GCPS when TMD pain is present and possibly prolonged.

The present study found no significant associations between TMD pain intensity (CPI) and parafunctions. These results are in contrast with some earlier findings. In the study of Khawaja *et al*. [10] focusing on waking-state parafunctional activities, the subjects with high pain intensity had higher mean OBC score than those with low pain intensity or no pain [9], and similar results were reported in the study of Donnarumma *et al*. [33] Additionally, in the study by Vrbanović *et al*. [19], oral behaviors were associated with pain intensity, but not with the presence of pain.

In this study, sleep bruxism was associated with pain widespreadness between distinct PD profile subgroups (PD-1 *vs.* PD-2 and PD-1 *vs.* PD-3), indicating that sleep bruxism was significantly less reported by patients with local pain as compared to those with regional (neck-shoulder area pain) or widespread pain. Mean OBC scores were also highest among patients with head and shoulder area pain (PD-2) and widespread pain (PD-3), differing significantly when comparing PD-1 and PD-2 OBC mean sum scores. The results can be explained by the close connection between masticatory muscles and neck-shoulder muscles [1]. Furthermore, in the study by Sahbaz *et al*. [34], tooth grinding and masseter hypertrophy were significantly more prevalent in fibromyalgia patients compared to healthy controls. The study by Huhtela *et al*. [12] from student population also found that self-reported bruxism is associated with widespread pain. The mechanism behind this association between bruxism and widespread pain may be linked to neurochemical and central regulatory mechanisms, creating a need for further studies.

Of the sociodemographic variables, marital status (single) was associated with both sleep bruxism and daytime clenching, and working status (employed) was associated with sleep bruxism. Female gender was associated with daytime clenching, although the number of patients in distinct subgroups remained low. Studies have found various, partly discrepant results concerning the sociodemographic factors in the background of bruxism. In a Dutch population, higher sociodemographic status and higher age were associated with bruxism [35]. Instead, in another population-based study [36] lower educational level was associated with awake bruxism among men. In other studies, there exist other potential background factors for bruxism that were not considered in this study, such as the use of nicotine and alcohol, and possibly, medications and addictive substances [37]. Some antidepressants, for example, may induce bruxism and may thus have affected the associations reported here.

The study population was based on a multi-center study on TMD pain patients. The strength of the present study is the use of DC/TMD Axis I and II questionnaires which have high sensitivity and specificity levels. Clinical examinations were performed by calibrated professional dental specialists according to the DC/TMD clinical protocol. The study was based on a large sample of TMD pain patients. PDs were analyzed by an independent evaluator without seeing the patient or other patient data, giving a more unbiased analysis, and PD findings have been shown to be in line with self-reported comorbid pain results [18]. The number of non-responses for the questionnaires was relatively low, except for the total OBC sum score. The possible bias due to non-response for OBC was analyzed and showed to be low. To assess larger sample sizes for statistical analysis considering OBC Q1–4 (sleep bruxism and daytime clenching) dichotomization for frequent *vs.* non-frequent parafunction activities during sleep and waking hours was done. However, as there exists no definite recommendation for the cutoffs for the presence or absence of bruxism, the OBC questions were also used as continuous variables, showing parallel associations. Two questions of the OBC were selected for the assessment of sleep bruxism and daytime clenching. For the latter, the question concerning teeth grinding during waking hours (Q3) was left out from the analyses because of the low number of reports. Instead, we used the question concerning tooth clenching (Q4), which was more frequently reported here, and which is also considered to be a typical form of daytime parafunction [38]. In future studies this factor may affect data integrity and must be taken into account. Furthermore, the small size of some subgroups, such as only 22 patients in TMD subtype 2, may have reduced the statistical power. This is a limitation of the study, and the results should therefore be interpreted with caution.

The lack of objective assessment for sleep bruxism is a limitation of the study. Self-reporting is inherently limited during sleep behaviors, as individuals are often unaware of their actions while asleep. This limitation underscores the need for objective measures in future studies, such as polysomnography or other sleep studies, to better understand and measure sleep bruxism.

## 5. Conclusions

Sleep bruxism and daytime clenching were associated with muscle-related TMD diagnoses and headache attributed to TMD, anxiety symptoms, and wider body pain distribution. Patients with moderate pain intensity/interference reported sleep bruxism most frequently. These results emphasize the role of biopsychosocial factors in the background of parafunctions among TMD pain patients, which should be considered in the assessment, treatment planning and personalized care of TMD pain patients.

In clinical practice, screening both parafunctions and psychosocial factors using DC/TMD instruments would facilitate both assessment and individualized patient care planning. Multidisciplinary treatment is recommended in the treatment scheme for TMD pain patients. Patient information is important in clinical practice for relieving of anxiety in TMD pain. Also, a referral to professionals in the field of psychologyas a part of the treatment is recommended in cases with high levels of psychological burden.

## 6. Highlights

 - Sleep bruxism was significantly associated with muscle-related TMD.

- Anxiety was significantly associated with both sleep bruxism and daytime clenching.

- TMD subtype associated significantly with frequent sleep bruxism; the highest proportion of sleep bruxism was observed in moderately compromised TMD subtype 2 patients.

- PD profile distribution associated significantly with sleep bruxism, regional PD-2 and widespread PD-3 subgroup patients reporting frequent sleep bruxism significantly more often than localized PD-1 subgroup patients.
